# Supplementary material for: Modeling drug combination effects via latent tensor reconstruction
Source: Bioinformatics. 2021 Jul 12;37(Suppl 1):i93–i101. doi: 10.1093/bioinformatics/btab308 (PMC8336593; doi:10.1093/bioinformatics/btab308)
Supplement: btab308_Supplementary_Data [file btab308_supplementary_data.pdf]

Modeling drug combination effects via latent tensor reconstruction

Supplementary matrials

March 26, 2021

1 Supplementary Figures

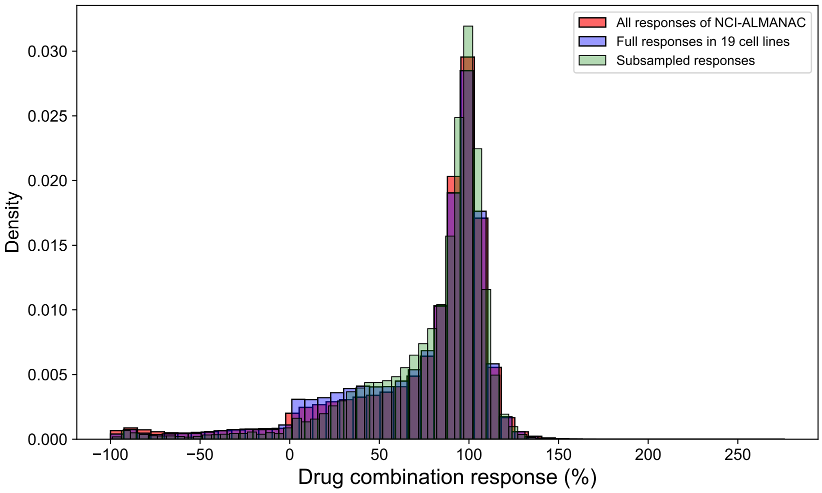

Figure S1: Distributions of drug combination responses of the whole NCI-ALMANAC data; full responses in 19 cell lines; and subsampled dataset.

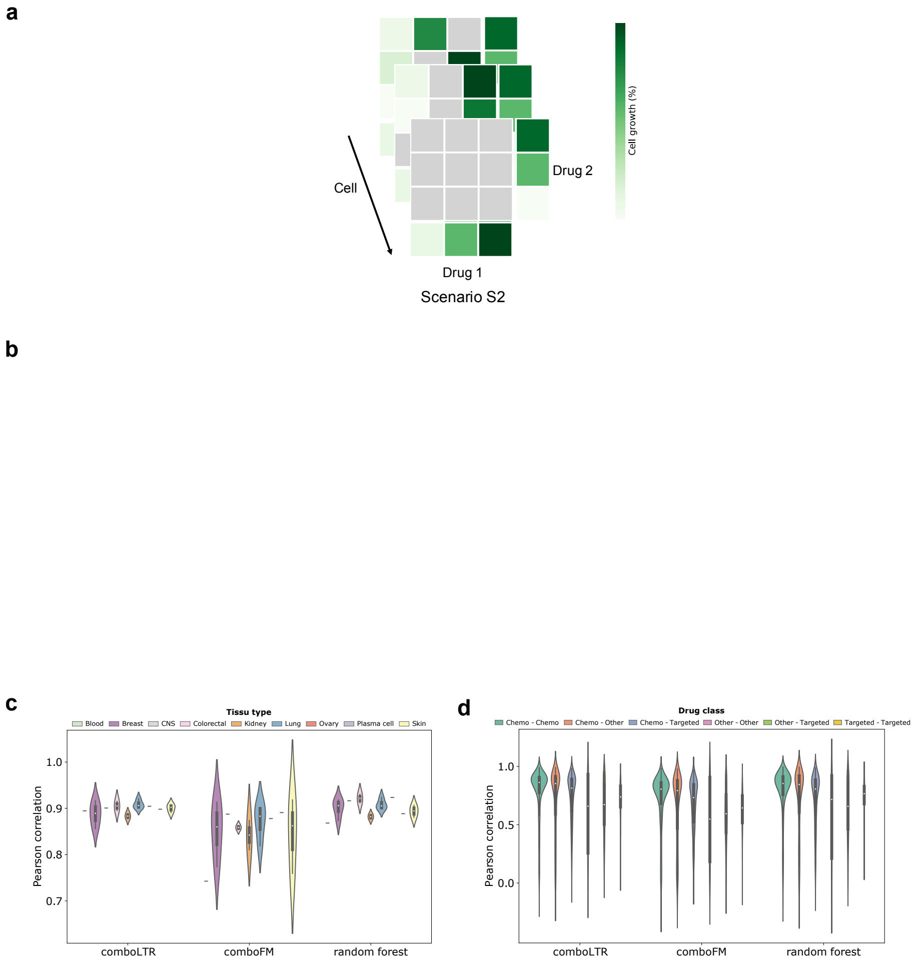

Figure S2: **(a)** illustration of prediction scenario: predicting dose-responses of previously untested drug-drug-cell line triplets; for each drug combination, the whole dose-response matrices were randomly selected into test sets, such that drug combination is still present in the training set but in other cell lines. **(b)** predictive performance of *comboLTR*, *comboFM* and random forest in the illustrated scenario: scatter plots of predicted and measured drug combination responses. Prediction performance of *comboLTR* across **(c)** tissue types and **(d)** drug classes: violin plots of Pearson correlations of predicted and measured drug combination responses in different tissue types and drug classes.

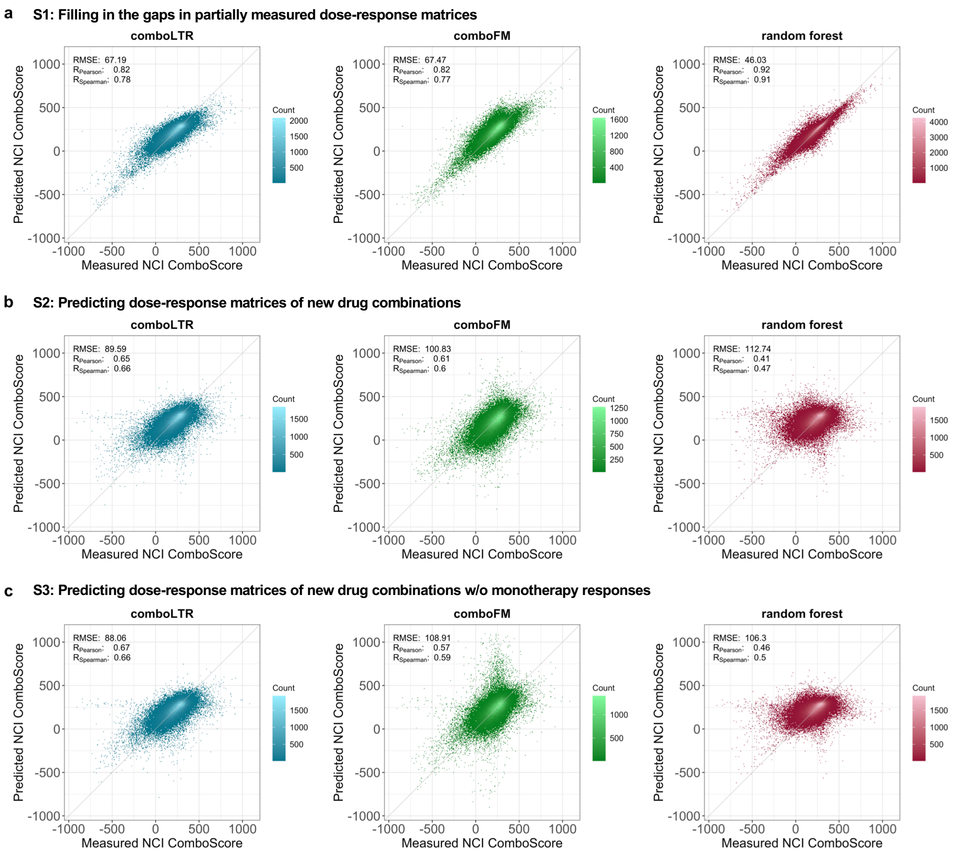

Figure S3: Predictive performance of *comboLTR*, *comboFM* and random forest in predicting NCI ComboScores in three prediction scenarios. Scatter plots between the NCI ComboScores calculated based on predicted and measured drug combination effects in the form of %-growth of cancer cell lines. The predictions were made under three scenarios of (a) filling in the gaps in partially measured dose-response matrices, inferring dose-response matrices of completely new drug combinations with (b) and without (c) monotherapy responses available. Diagonal line is displayed in each scatter plot.

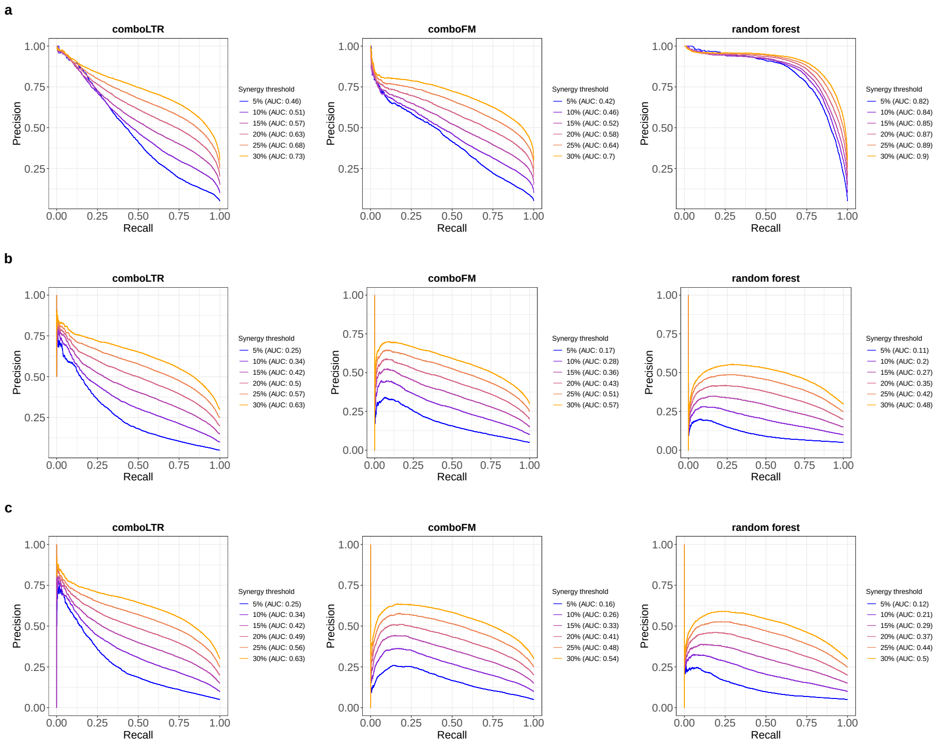

Figure S4: Precision-recall (PR) curves for *comboLTR*, *comboFM* and random forest. PR curves were used to evaluate the model performance in classifying drug combinations as synergistic vs. non-synergistic with varying thresholds for synergy, in the three prediction scenarios: (a) filling in the gaps in partially measured dose-response matrices, inferring dose-response matrices of completely new drug combinations with (b) and without (c) monotherapy responses available. Area under the PR curve (AUPR) is shown in parenthesis.

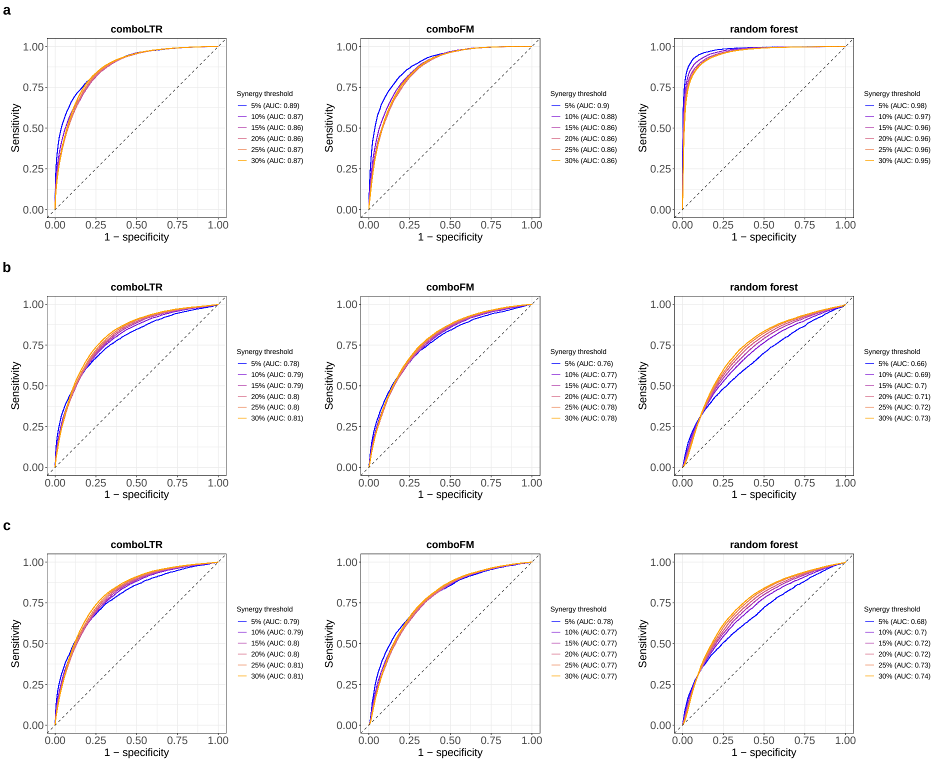

Figure S5: Receiver operating characteristic (ROC) curves for *comboLTR*, *comboFM* and random forest. ROC curves were used to evaluate the model performance in classifying drug combinations as synergistic vs. non-synergistic with varying thresholds for synergy, in the three prediction scenarios: (a) filling in the gaps in partially measured dose-response matrices, inferring dose-response matrices of completely new drug combinations with (b) and without (c) monotherapy responses available. Area under the ROC curve (AUC) is shown in parenthesis.

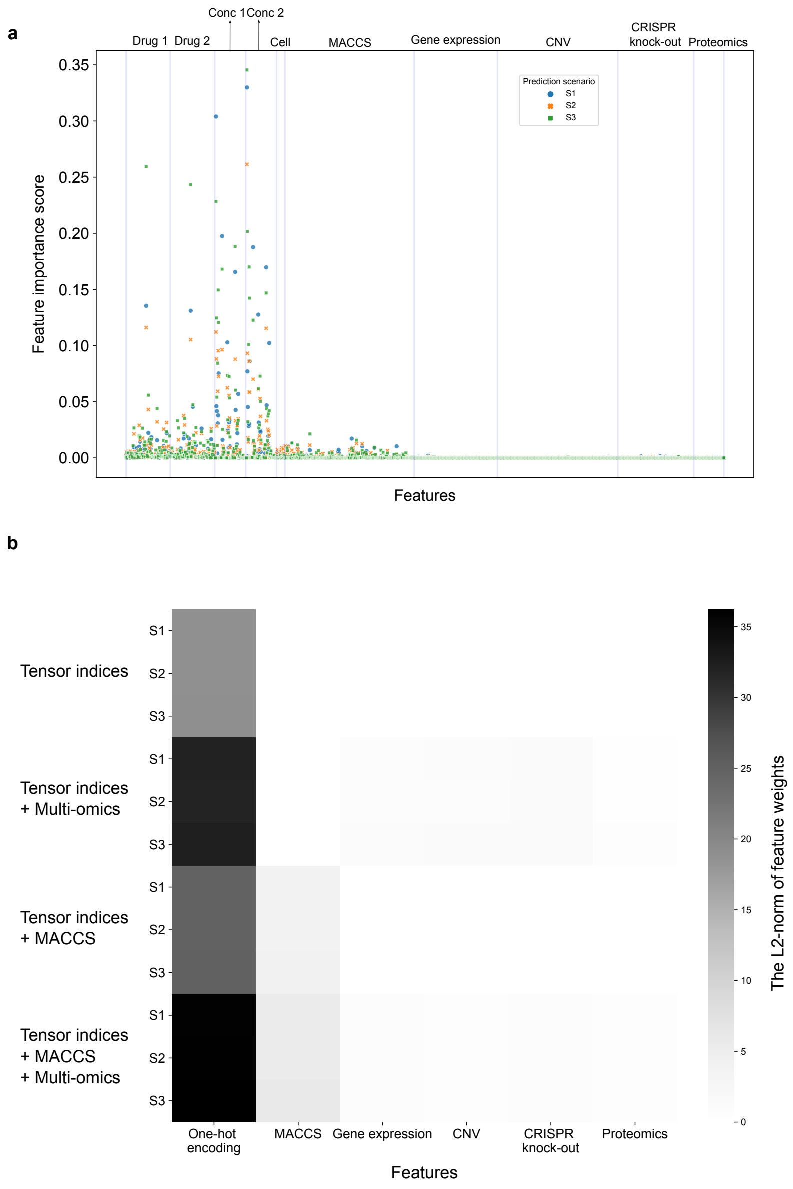

Figure S6: **(a)** feature permutation importance score of each feature; calculated as the difference of Pearson correlations before and after randomly permuting a feature of all samples. **(b)** *comboLTR* feature weights in different feature combinations; the L2-norm of the weights of each feature set was used to measure the importance of that feature set.

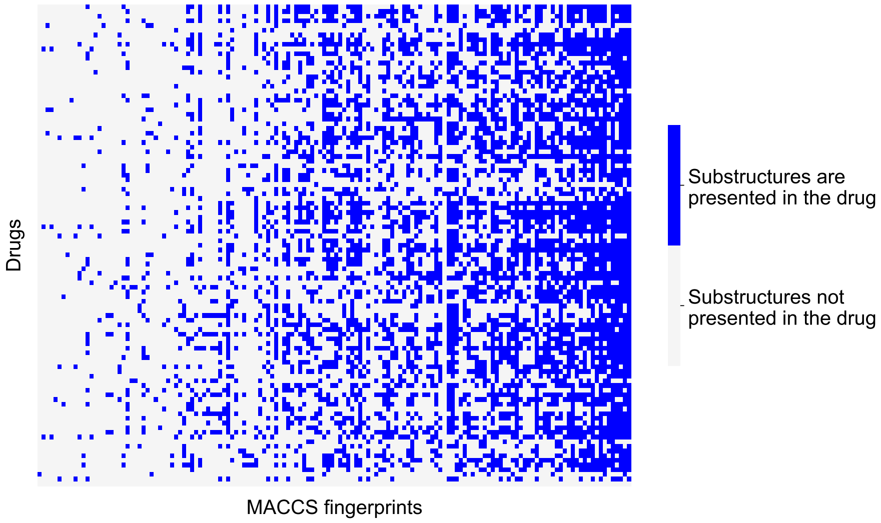

Figure S7: Heatmap of MACCS fingerprints across different drugs. Substructures defined by MACCS fingerprint were matched with drug chemical structures.

2 Supplementary Tables

Table S1: *comboLTR*, *comboFM*, and random forest Pearson correlations between predicted and measured drug combination responses for prediction scenario: predicting dose-responses of previously untested drug-drug-cell line triplets.

Feature combination	*comboLTR*	*comboFM*	random forest
Tensor indices	0.897±0.007	0.904±0.007	0.898±0.008
Tensor indices + MACCS	0.903±0.006	0.898±0.045	0.899±0.009
Tensor indices + Multi-omics	0.867±0.012	0.884±0.031	0.888±0.007
Tensor indices + MACCS + Multi-omics	0.901±0.006	0.857±0.046	0.901±0.006
